# Active Trachoma Prevalence and Related Variables among Children in a Pastoralist Community in Southern Ethiopia in 2021: A Community-Based Cross-Sectional Study

**DOI:** 10.4269/ajtmh.22-0521

**Published:** 2023-01-09

**Authors:** Dedefo Tuke, Edao Etu, Endale Shalemo

**Affiliations:** ^1^ The Fred Hollows Foundation Ethiopia, Nagele, Ethiopia;; ^2^ Department of Public Health, Madda Walabu University, Shashemene, Ethiopia

## Abstract

An estimated 30% of trachoma burden is borne by Ethiopia. Data on the prevalence of active trachoma and related factors in a pastoralist population are currently lacking. Additionally, no research has been conducted in the Oromia, Guji Zone of the Liben District. A community-based cross-sectional study was conducted among 538 children 1–9 years old in the pastoralist community of the Liben District from March 1 to April 30, 2021. A multistage systematic sampling method was applied to choose the sample. A structured questionnaire and WHO’s trachoma grading scheme were used to identify active trachoma. Bivariate and multivariable logistic regression models were fitted to determine associated factors. An adjusted odds ratio with 95% confidence interval was calculated to decide the level of significance: 157 (29.2%) (95% CI: 24.9, 33.1) of children had clinical signs of active trachoma, 103 (66%) had trachomatous follicles, 41 (26%) had trachomatous intense, and 13 (8%) had both. There was an independent relationship between active trachoma and open defecation (adjusted odds ratio [AOR]: 2.75; 95% CI: 1.24, 6.09), defecating outside close to a house (AOR: 2.5; 95% CI: 1.07, 6.08), not having a latrine (AOR: 3.70; 95% CI: 1.60, 8.60), children who did not wash their faces with soap (AOR: 1.85; 95% CI: 1.10, 3.07), and being in a widowed household (AOR: 3.26; 95% CI: 1.57, 6.63). The study’s findings revealed that about one-third of the children had clinical signs of trachoma. Research indicates that trachoma is a major concern for children in rural communities. Therefore, attention to trachoma control with antibiotics, facial hygiene, and environmental sanitation is strongly encouraged.

## INTRODUCTION

A *Chlamydia trachomatis* bacterial infection causes trachoma, the most prevalent infectious eye disease. It is the most common infectious cause of blindness in the entire world. Follicles on the tarsal conjunctiva are a result of this bacterial infection, which also manifests as redness, irritation, and sensitivity to light.^1^ Hand-to-eye contact, mosquitoes, or flies searching for human eyes are the main methods of transmission for this illness.^2^^,^^3^ *Chlamydia trachomatis* infection causes “active trachoma,” inflammatory conjunctivitis. The interior of the eyelid may suffer serious damage from recurrent infections, which can turn the eyelashes inward and cause them to rub against the cornea or eyeball. This extremely painful condition can result in corneal opacity, impaired vision, and blindness.^4^^–^^6^

The WHO lists the five stages of trachoma as trachomatous inflammation follicular (TF), trachomatous inflammation intense (TI), trachomatous scarring, trachomatous trichiasis (TT),^7^ and corneal opacity.^1^^,^^7^^,^^8^ Trachoma can result in total blindness or significant visual impairment. It can cause pain and disruptions for the people affected, their relatives, and their communities, as well as school dropouts, economic loss, and a deterioration in the quality of life.^9^^,^^10^ In locations where trachoma is endemic, surgery, antibiotics, facial cleanliness, and environmental hygiene techniques are being used for prevention and control.^3^^,^^11^

According to the WHO report of 2020, in 44 nations around the world, trachoma affects the public’s health. For roughly 1.9 million people, it is the cause of blindness or visual impairment. Trachoma poses a danger of blindness to 137 million individuals who reside in endemic areas.^4^ Around 1.4% of blindness in the world is a result of it. Africa continues to be the continent with the most severe effects and the one that is subject to the most intense control measures.^4^^,^^11^

**Figure 1.**
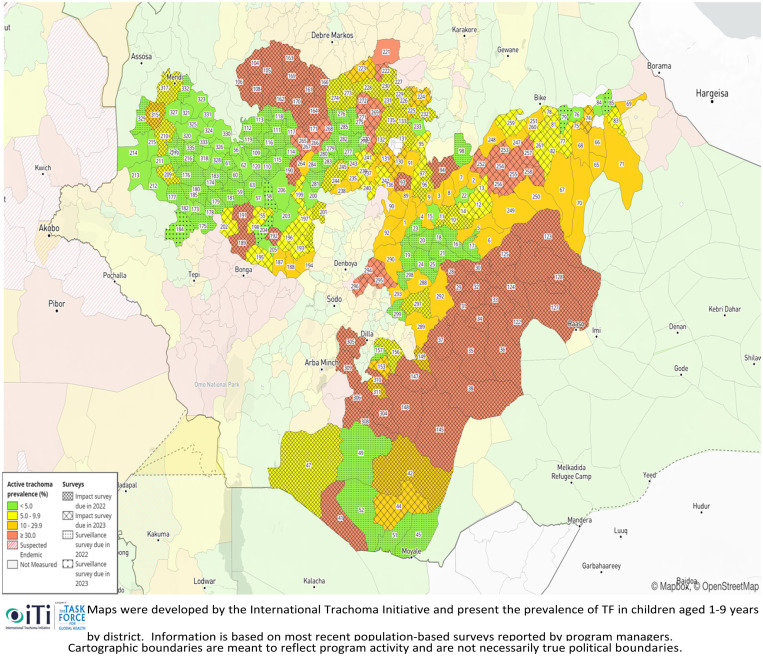
Active trachoma prevalence in Oromia, Ministry of Health data, 2020.

In Ethiopia, trachoma is the second-leading cause of blindness. More than 76 million persons are living with trachoma in widespread parts of the country. Over 9 million children aged 1–9 years live with active trachoma, and 1.3 million people 15 years and older have TT.^6^ In Oromia, the Global Trachoma Mapping Project (GTMP) findings of active trachoma in children aged 1–9 years showed that a population of over 27 million live in confirmed endemic areas (Figure 1). The prevalence of active trachoma was 23.4%.^9^ Studies conducted in other areas of Ethiopia similarly presented a high prevalence, including 21.5% in Deguatemben, Tigray, 15.2% in the Lemo District, southern Ethiopia, 22.5% in the Gonji Kolella District, West Gojjam, and 36.7% in the Zala District, Gamo Gofa Zone. These studies have contributed to our understanding of many aspects of the trachoma problem, including the magnitude of trachoma, risk factors, and effectiveness of control measures,^9^^,^^10^^,^^12^ and have emphasized the necessity for continued measures to control trachoma.^5^^,^^10^^,^^21^

There has not previously been a study providing information on trachoma in the Oromia region of the Liben District. Therefore, a cross-sectional study was carried out to ascertain the prevalence of active trachoma and associated factors in children from this region.

## MATERIALS AND METHODS

### Ethical considerations.

The Madda Walabu University Institutional Review Board provided ethical clearance. Written field permits were obtained from the Oromia Regional Health Bureau, Guji Zone Health Department, and Liben District Health Office. The study participants were asked for their assent orally and verbally. By excluding names from the questionnaire and respecting their privacy while being observed, the information was kept confidential throughout. Two tubes of tetracycline eye ointment were given to children with active trachoma for a period of 6 weeks.

### Study design, period, and setting.

In the pastoralist settlements of the Liben rural district, children between the ages of 1 and 9 years old were included in a community-based cross-sectional study from March 1 to April 30, 2021. The Guji Zone in the southern Ethiopian region of Oromia National Regional State includes Liben as one of its pastoralist rural districts. The distance between Addis Ababa (the capital city of Ethiopia) and Nagele Town, the district’s capital, is about 600 km. It has 12 kebeles (lowest administrative units). The district has 95,886 in total population, of which 48,518 are male and 47,368 are female, according to the Liban Woreda population forecast for 2020.^13^ The woreda has a total of 19,976 households.^13^ Nineteen health posts and three health centers are present. Health posts and health centers cover 65% of the same infrastructure, respectively. There is no ophthalmic professional in the district except for only one trained integrated eye care worker (IECW).

The study area experiences a rainy time of year between April and August and a dry period between September and March. During the dry season, the area is very hot and dusty. The community largely practices nomadic pastoralism as their primary source of income. The community lives in villages that consist of cow dung and mud huts with thatched roofs that are predominantly built by women. Livestock are mostly goats, cattle, and camels. The male children are mostly involved in taking care of the livestock whereas the females are mostly involved in household tasks.

The source population consisted of all children 1–9 years old in the Liben District of the Guji Zone. Randomly designated children 1–9 years old from selected households who fulfilled the inclusion criteria comprised the study population. Children aged 1–9 years old who lived for at least 1 month in the Liben District were included in the study. Children who had an eye injury and those who could not undergo physical examination because of their terminal illness were excluded from the study.

### Sampling techniques and subjects.

The first population proportion formula was used to determine the sample size for the first aim:

$$n={\left(Z\text{α}/2\right)}^{2}*p\left(1-p\right)/d^{2}.$$

According to the last study, the prevalence of active trachoma in children 1–9 years old was 35.5% (*P* = 0.355), where *p* = proportion of active trachoma (TF) among children 1–9 years old,^9^ *Z*/2 = confidence level, and *d* = desired precision level (margin of error). Assuming a 95% CI (= 0.05), 5% error margin, and *n* = (*Z*/2)^2^ * *p*(1 − *p*)/*d*^2^, *n* = (1.96)^2^ * 0.355(1 − 0.355)/(0.05)^2^ = 3.8416 * 0.228975/0.0025 = 352 and *n* = 387 when a 10% nonresponse rate is taken into consideration.

The sample size for the second objective was calculated using two population formulas in Epi Info version 7.2.2. From previous studies, the major associated factors for trachoma were the presence of a latrine, existence of a waste disposal pit, soap use for children’s face washing, and family size.^12^^,^^14^ These variables were selected based on the level of association and the narrower confidence interval to produce a large sample size. In this study, the solid waste disposal pit was the variable used to calculate the sample size with an estimated % outcome in the unexposed group of 5.3%, % outcome in the exposed group of 14.9%, and adjusted odds ratio (AOR) 3.1, based on 95% CI and 80% power; a ratio of unexposed to exposed is 1.6.

Accordingly, the sample size for the two exposed and unexposed groups was 354. Adding 10% for possible nonresponse rate, a minimum sample size of 389 was needed. By comparing the sample size calculated for both objectives, the maximum sample size of 389 calculated for the second objective was taken. After the application of finite population correction and multiplying by the design effect of 1.5, the final sample size for this study was 540.

Participants were chosen from kebeles in the Liben area using multistage random sampling techniques. Four kebeles were randomly chosen from among the 12 in the Liben Woreda (lottery method). Siminto, Kobadi, Arda Bururi, and Karsamale kebeles were ultimately chosen. The mass drug administration (MDA) registers, which were compiled by health extension workers through a house-to-house regular visitation program in specified kebeles, contained lists of households with children aged 1–9 years old, which were used to create the sampling frame. Then, it was decided how many homes with children ages 1–9 would be sampled from each kebele based on the relative distribution of children aged 1–9 in each kebele. Following that, the sample size and sampling interval were calculated from the number of households with children aged 1–9 in each kebele. Then, homes with at least one child between the ages of 1 and 9 were chosen using a systematic random sampling technique. Using a lottery, the first household was chosen. Finally, one child was chosen at random (lottery) for homes with more than one child aged 1–9 years. Screening for active trachoma was conducted on a selected household’s 1- to 9-year-old child.

### Study variables.

#### Dependent variable.

The study dependent variable was the presence of active trachoma, which meant the existence of TF and or TI, which was identified by trained IECWs.

#### Independent variables.

The independent variables for this study were categorized under the following factors: sociodemographic factors that included respondents’ and children’s age, children’s age measured as 1–4 and 5–9 years, respondents’ and children’s sexes, education status of both parents and children, marital status of the head of the household (which was measured as married, divorced, and widowed), and occupation of the head of the household.

Household factors comprised the main source of water measured by the family (piped water, protected or unprotected springs, rainwater, and surface water); time to fetch water as the time taken to fetch water and come home for one trip, measured as < 15, 15–30, 30–60, and > 60 minutes; living with livestock; number of children living in the household, measured as < 5 and ≥ 5; and family income. Knowledge about trachoma was measured by using 14 questions that assessed respondents’ knowledge about trachoma and categorized knowledgeable and poor knowledge using the appropriate measure (mean) of central tendency.

Individual child hygiene factors are frequency of face washing, measured as more than once per day, one time per day, and only occasionally, and use of soap for face washing as measured by using or not using soap to wash the child’s face. Environmental sanitation factors include the availability of latrines, availability of handwashing facilities, solid waste disposal, and liquid waste disposal, all confirmed as present or absent by observation.

### Operational definition.

Active trachoma is the existence of TF and or TI, which was identified by trained IECWs.^4^ Trachomatous inflammation follicular is the presence of at least five or more follicles in the upper tarsal conjunctiva, each at least 0.5 mm in size.^4^ Trachomatous inflammation intense is an inflammatory thickening of the upper tarsal conjunctiva with more than 50% of deep tarsal vessels obscured.^4^ Time to fetch water is the time taken for one journey to fetch water and come back home.^11^ A clean face is defined as a child who did not have an eye or nasal discharge or a fly in the face at the time of observation. Knowledge about trachoma was calculated by 14 different queries on signs and symptoms, transmission modes, causes and consequences of trachoma, and prevention and control measures of trachoma and by coding the responses as 1 for correct answers and 0 for incorrect answers and adding all the values. An appropriate measure of central tendency used for categorizing data was determined based on the shape of the distribution. Then, a respondent who scored above or equal to the mean was labeled as knowledgeable and those who scored below the mean were labeled as having poor knowledge.^15^ Open defecation is the emptying of bowels in the open bushes, which is also outside of their compounds, without the use of properly designed structures built for the handling of human waste such as toilets.^11^

#### Data collection procedure and analysis.

The study used a quantitative data collection method.

#### Interview.

There were five sections to the study interview. Part 1 covered sociodemographic variables, part 2 covered household information, part 3 covered environmental sanitation, part 4 covered child-specific hygiene, and part 5 covered knowledge about trachoma. The household was surveyed by the data collectors (health extension workers; HEWs), and the small children were evaluated by the IECWs. To locate the heads of households with their children in the specified residence, the examinations and interviews were set for the early morning hours.

#### Observation.

Direct observation was used to check the occurrence of ocular/nasal discharge on the child’s face, presence of a fly on his/her face within 3 seconds of observation, signs of active trachoma, and presence of a turned-in eyelash. Availability and utilization of a latrine, area of defecation for adults in the household, availability of a garbage pit, and environmental sanitation were also observed.

#### Eye examination.

Using a binocular loupe with convergent magnification (2.5×) and a hand flashlight, two senior IECWs who had completed the customary 2-day training using the GTMP training manual^9^ and WHO guidelines examined the child’s eyes. Each eye was examined closely, with special focus being placed on the cornea, top lid eversion, and eyelash development, examination of the tarsal conjunctiva, and recording of the findings. When both eyes were affected during an eye exam, the results of the worse eye were noted. The WHO’s streamlined grading approach was properly followed during the eye exam and trachoma grading procedures were used.^1^ After each examination the required hygiene precautions were also performed, such as washing hands with an alcohol-based hand gel, to avoid the spread of illness among subsequent participants. Clinical symptoms that were required to be regarded as present were readily apparent. A sign was noted as lacking when there was uncertainty.

Data were gathered using a pretested, structured questionnaire (Afan Oromo version) that was developed from many academic works. Data gatherers used an observation checklist. Trachoma grading used the standard WHO’s streamlined trachoma classification system.^1^ Senior IECWs examined children’s eyes using a pair of binocular loupes with convergent (2.5×) magnification and a torch-style hand light. Two ophthalmic nurses who worked in the neighborhood and two HEWs who would gather data were also hired for monitoring and eye exams. For 2 days, training was provided to data gatherers so they would be familiar with the equipment. Additionally, the researcher managed the daily coordination of all data collection activities.

To control quality, the study strived to attain validity and reliability. The questionnaire was first developed in English, translated into Afan Oromo (the local language), and backtranslated to English by language experts to check its consistency. The questionnaires developed were pretested using 10% of the sample size before the main study was conducted. Based on the pretest, questions were assessed for their clarity, length, and completeness, and those found to be unclear were corrected. Data quality was also ensured by training data collectors and supervisors as well as providing day-to-day supervision during the whole period of data collection. The research supervisors were trained IECWs and data collectors were trained HEWs because the participants were familiar with these individuals and were very likely to volunteer truthful information to them. The supervisors and data collectors also knew both spoken and written English and the local dialect. The principal investigator checked completed questionnaires for errors and corrections made before entry into a computer software program for analysis.

The accuracy and consistency of the data were verified before being coded, entered, and exported to SPSS (Statistical Package for the Social Sciences) version 25 (Chicago, IL) for analysis using Epi Info version 7.2. For each variable, a univariate analysis was conducted using frequencies and percentages. The related factors of the outcome variable were found using binary logistic regression analysis. Covariates with *P* values of 0.25 in the bivariable analysis were taken into consideration as candidates for the multivariate analysis. The adequacy of the data for multiple logistic regression was examined using Hosmer and Lemeshow’s goodness-of-fit test. To identify the independent factors of active trachoma, a multivariable logistic regression model was used. AOR was used to interpret the final model at a *P* value of 0.05 and a 95% CI.

## RESULTS

### Sociodemographic characteristics of study participants.

538 of 540 children (aged 1–9 years) were examined for trachoma, with a response rate of 99.6% (Table 1). Of the 538 households interviewed, 301 (55.9%) respondents were females; 189 (35%) of the participants were aged between 30 and 40 years, with the mean age of the respondent being 2.4 years with SD 1.2. Greater than two-thirds of the respondents were married 443 (82.3%) and 52 (9.6%) were widowed. The majority, 499 (92.8%), of the respondents were farmers. Almost one-half of the children, 272 (50.6%), were aged between 1 and 4. There were 243 preschool children (45.2%).

**Table 1:** Sociodemographic information about research participants in the southern Ethiopian region of Guji Zone’s Liben District, July 2021

Variable		Frequency	Percentage
Age of respondents (years)	19–29	131	24.2
	30–40	189	35.0
	41–50	133	24.6
	51–60	54	10.0
	≥ 61	33	6.1
Sex of respondents	Male	237	44.1
	Female	301	55.9
Marital status of respondents	Married	443	82.4
	Divorced	43	8.0
	Widowed	52	9.6
Educational status of respondents	Illiterate	381	70.7
	Can read and write	93	17.6
	Elementary and above	64	11.7
Occupation of head of household	Farmer	499	92.8
	Others	39	7.2
Age of child (years)	1–4	272	50.6
	5–9	266	49.4
Sex of child	Male	221	41.1
	Female	317	58.9
Educational status of child	Too young to enroll	243	45.2
	Not enrolled	162	30.2
	Dropped out of school	27	5.0
	Attending school	106	19.6

### Household characteristics of respondents.

Two hundred and ten (39%) households had more than five children aged 1–9 years in residence (Table 2). The public water sources for local consumption in the study area were rainwater collection for 293 (54.6%) of the study children. Only 104 (19.3%) of respondents got their water as piped water. One hundred and eighty-three (33.9%) of households spent more than 60 minutes collecting water. The estimated average monthly income of 258 (48%) households was less than 600 Ethiopian birr. The mean family income was 1.9 with SD 0.044.

**Table 2:** Household and knowledge factors of study participants in Liben District, Guji Zone, southern Ethiopia, July 2021

Variable		Frequency	Percentage
Number of children living in household	< 5	328	61.0
	≥ 5	210	39.0
Average monthly income (ETB)	< 600	258	48.0
	601–1,200	134	24.9
	1,201–2,000	88	16.4
	> 2,000	58	10.7
Main source of water for family	Piped water/public tap	104	19.3
	Protected well/spring	41	7.6
	Unprotected well/spring	20	3.7
	Rainwater collection	293	54.6
	Surface water (river/pond)	80	14.8
Time to fetch water (minutes)	< 15	100	18.6
	16–30	144	26.9
	31–60	111	20.6
	> 60	183	34.0
Living with animals	No	484	89.6
	Yes	54	10.4
Knowledge about trachoma	Knowledgeable	405	75.3
	Poor knowledge	133	24.7

### Knowledge about trachoma.

The majority, 405 (75.3%), of the respondents were knowledgeable (Table 2). Only 133 (24.7%) of the participants had poor knowledge about trachoma.

### Environmental sanitation factors of participants.

Adults in the majority of households, 346 (64.3), used latrines to defecate (Table 3). One hundred and twenty-three (23%) of the families used open defecation. In the study area, 236 (43.9%) of families had no latrine. The majority, 398 (74%), of study children were from families who had no handwashing facilities. In the study area, 188 (35%) of families disposed of their domestic wastes in an open field.

**Table 3:** Environmental sanitation factors of study participants in Liben District, Guji Zone, southern Ethiopia, July 2021

Variable		Frequency	Percentage
Defecation area for adults in household	Use latrine	346	64.3
	Outside near house	69	12.8
	Open defecation	123	23.0
Availability of latrine	No	236	43.9
	Yes	302	56.1
Availability of handwashing facilities	No	398	74.0
	Yes	140	26.0
Means of garbage disposal	Burning	335	62.2
	Drying	15	2.8
	In open field	188	35.0

### Child hygiene factors.

Two hundred and forty-seven (45.9%) of the study children washed their faces only occasionally (Table 4). Only 132 (24.6%) of the study children washed their faces more than one time per day. Two hundred and thirty-nine (44.4%) of the study children did not use soap for face washing.

**Table 4:** Individual child hygiene factors of study participants in Liben District, Guji Zone, southern Ethiopia, July 2021

Variable		Frequency (*n* = 540)	Percentage
Frequency of face washing	More than once per day	132	24.5
	One time per day	159	29.6
	Only occasionally	247	45.9
Use of soap during face washing	No	239	44.4
	Yes	299	55.6
Facial cleanliness of the child	No	237	44.1
	Yes	301	55.9

### Prevalence of active trachoma.

Out of the 538 children aged 1–9 years examined, active trachoma was observed in 157 (Table 5).

**Table 5:** Prevalence of active trachoma among children aged 1–9 years in Liben District, Guji Zone, southern Ethiopia, July 2021

Variable		Frequency (*N* = 157)	Percentage	Interval	
Stage of trachoma	TF	103	19.1	15.6	22.6
	TI	41	7.6	5.4	9.8
	Both	13	2.4	–	–

TF = trachomatous follicular; TI = trachomatous intense.

The prevalence of active trachoma in rural communities of the Liben District was 29.2% (95% CI: 24.9, 33.1). Of these, 19.2% were trachomatous inflammation intense, 7.6% were trachomatous inflammation follicles, and 2.4% of the examined children had the combination of both stages.

### Determinants of the frequency of active trachoma.

In a multivariate analysis, characteristics that were independently associated with active trachoma in children aged 1–9 years were marital status of parents, access to latrines, location of adults’ places of defecation, and use of soap for face washing. The presence of active trachoma was highly linked with the marital status of the head of the household. Children of widowed mothers were 3.2 times more likely to have active trachoma than children of married mothers (AOR: 3.20; 95% CI: 1.56, 6.60) (Table 6). Active trachoma was around 4 times more prevalent in children from families without latrines than in their peers (AOR: 3.70; 95% CI: 1.60, 8.50). Children from families who defecated outside close to the house were 2.5 times more likely than their peers to acquire active trachoma (AOR: 2.5; 95% CI: 1.1, 6.03). Significant correlations were found between the use of soap for face washing and active trachoma. Children who did not wash their faces with soap had an almost 2-fold increased risk of developing active trachoma compared with those who did (AOR: 1.9; 95% CI: 1.13, 3.12).

**Table 6:** Summary of the factors that are connected to active trachoma in children aged 1–9 years in Liben District, Guji Zone, southern Ethiopia, July 2021

Variable	Active trachoma			Crude OR (95% CI)	Adjusted OR (95% CI)
	Category	Yes (*N* = 157)	No (*N* = 383)		
Marital status	Married	105 (66.9)	338 (88.7)	1	1
	Divorced	20 (12.7)	23 (6)	2.82 (1.49, 5.3)	1.40 (0.64, 3.0)
	Widowed	32 (20.4)	20 (5.2)	5.2 (2.84, 9.4)	3.20 (1.56, 6.60)**
Educational status	Illiterate	122 (77.7)	259 (68)	2.57 (1.27, 5.22)	–
	Can read and write	25 (15.9)	69 (18.1)	1.9 (0.80, 4.14)	–
	Elementary and above	10 (6.4)	53 (13.8)	1	–
Age of child (years)	1–4	67 (42.7)	205 (53.8)	1	–
	5–9	90 (57.3)	176 (46.2)	1.56 (1, 2.27)	–
Number of children in household	< 5	77 (49)	251 (65.8)	1	–
	> 5	80 (51)	130 (34.2)	2 (1.4, 2.9)	
Main source of water	Piped water/public tap	7 (4.5)	97 (25.5)	1	–
	Protected well/spring	10 (6.4)	31 (8.4)	4.47 (1.6, 12.7)	–
	Unprotected well/spring	7 (4.5)	13 (3.4)	7.5 (2.25, 24.7)	–
	Rainwater collection	94 (59.9)	199 (52.2)	6.5 (2.9, 14.5)	–
	Surface water (river/pond)	39 (24.8)	41 (10.7)	13.2 (5.4, 31.9)	–
Time to fetch water (minutes)	< 15	21 (13.4)	79 (20.9)	1	–
	16–30	25 (15.9)	119 (31.3)	0.79 (0.42, 1.5)	–
	31–60	41 (26.1)	70 (18.3)	2.2 (1.2, 4.1)	–
	> 60	70 (44.6)	113 (29.5)	2.36 (1.3, 4.1)	–
Living with animals	Yes	28 (17.8)	28 (7.3)	2.75 (1.57, 4.82)	1.94 (0.94, 4.0)
	No	129 (82.2)	353 (92.7)	1	1
Defecation area	Use latrine	39 (24.8)	307 (80.6)	1	1
	Outside near house	42 (26.8)	27 (7)	12.29 (6.83, 22.09)	2.5 (1.10, 6.03)**
	Open defecation	76 (48.4)	47 (12.4)	12.5 (7.65, 20.4)	2.8 (1.27, 6.22)**
Availability of latrine	Yes	26 (16.6)	276 (72.3)	1	1
	No	131 (83.4)	105 (27.7)	12.67 (7.9, 20.4)	3.7 (1.60, 8.50)**
Availability of handwashing facilities	Yes	10 (6.4)	130 (33.9)	1	–
	No	147 (93.6)	251 (66.1)	7.55 (3.85, 14.83)	–
Frequency of face washing	More than once per day	13 (8.3)	119 (31.3))	1	1
	One time per day	28 (17.8)	131 (34.2)	1.99 (0.98, 3.99)	0.74 (0.32, 1.69)
	Only occasionally	116 (73.9)	131 (34.5)	8.1 (4.3, 15.1)	1.4 (0.62, 3.2)
Use of soap during face washing	Yes	49 (31.2)	250 (65.5)	1	1
	No	108 (68.8)	131 (43.5)	4.2 (2.8, 6.24)	1.9 (1.13, 3.12)**
Knowledge about trachoma	Knowledgeable	102 (65)	303 (79.5)	1	–
	Poor knowledge	55 (35)	78 (20.5)	2.1 (1.4, 3.2)	–

** = represents variables or categories of a variable significantly associated with active trachoma (based on *P* value and AOR). OR = odds ratio. Bivariate and multivariable logistic regression analysis.

## DISCUSSION

In the research area, active trachoma was reported to be present in 29.2% (95% CI: 24.9, 33.1) of children aged 1–9. According to the results of the current study, the prevalence of active trachoma was higher than the WHO target of 5% for trachoma eradication in children between the ages of 1 and 9 years. The GTMP between 2012 and 2014 reported that the prevalence of TF in the districts of Liben, Adola Rede, Odo Shakiso, and Wadera was 35.5%; however, the current finding was lower than that number.^9^ According to the findings, the prevalence of active trachoma was also lower in the Liben District compared with studies done in the Zala District, southern Ethiopia, 2016; Areka Town, south Ethiopia, 2018; and Gazegibela District of Wagehemra Zone, northern Ethiopia, 2016 (52.4%).^16^^–^^18^ The full implementation of the SAFE (surgery, antibiotics, facial cleanliness, and environmental improvement) approach’s F and E mechanisms, along with the woredas’ MDA of azithromax and tetracycline eye ointments in the Liben area, may be one explanation for this divergence. Other possible explanations for this discrepancy are that the sample size was not the same, the endemicity was different, and there was a time difference. This study was carried out after the GTMP survey, when significant efforts had already been undertaken in Oromia State to control trachoma. This suggests that the WHO-endorsed SAFE method should be implemented to prevent, control, and eliminate blinding trachoma.

The prevalence of active trachoma in the Liben District was largely consistent with studies carried out at the district level in other parts of the country, such as the survey carried out by GTMP in the Arsi Zone (29.6%), Borena Zone, 2016 (29.2%), and Grar Jarso, 2018 (27.8%).^1^^,^^2^ The study findings showed that the frequency of active trachoma in this study area was also similar to studies conducted in Kenya, 2019 (27%).^3^

The prevalence of active trachoma in the current study was higher than in studies done in the Madda Walabu District in 2019 (21.9%), Loma Woreda, Dawro Zone in 2015 (22.85%), Baso Liban District, East Gojjam in 2012 (24.3%), Deguatemben District, Tigray in 2018 (21.9%), Mojo and Lume Districts of Ethiopia in 2012 (22.51%), and Kajiado County of Kenya in 2020 (15.7%).^4^^–^^7^^,^^14^^,^^24^ The higher prevalence in this study might be due to the endemicity of the disease in the district, lower coverage of water and sanitation facilities, climatic conditions, and living conditions of the study area population. The prevalence was also beyond the WHO benchmark definition of public health importance. This implies that the finding of the current study confirmed that trachoma is still a disease of public health importance in the Liben District.

This study showed that the children of widowed household heads were 3.2 times more at risk of having active trachoma as compared with married household heads (OR: 3.3; 95% CI: 1.57, 6.7; *P* = 0.001). In agreement with this study, a study conducted in the Oromia Regional State of Ethiopia in 2016 showed that widowed households are 1.3 times more likely to have active trachoma than married households.^1^ The possible reason for this difference may be that the economic burden, responsibility of caring for children, and social burden were only on the wife/husband. This implies that trachoma is a disease of poverty and widowed households may be more likely to be experiencing poverty, which exposes them to failure to implement preventive measures.

According to the findings of the current study, children who lived in households without latrines were 3.7 times more likely to experience active trachoma than those from households with latrines (AOR: 3.7; 95% CI: 1.6, 8.6). The studies conducted in Gonder Zuria, northern Ethiopia, 2015 (OR: 2.0; 95% CI: 1.34, 7.6), Madda Walabu District, southeast Ethiopia (OR: 2.5; 95% CI: 1.8, 5.3), and Dembiya District, northwest Ethiopia (OR: 1.6; 95% CI: 1.0, 2.5) that showed a significant association between latrine availability and active trachoma were in line with this finding.^4^^,^^8^^,^^9^ In addition, it was also similar to the study conducted in Kenya.^10^ This might be justified in that inaccessibility to latrine facilities would result in exposure to human feces, which was the risk factor for the presence of a high number of flies that leads to a higher chance of transmission of active trachoma. This implies that the existence and utilization of a latrine at the family level may decrease eye-seeking flies in the adjacent setting.

However, the study findings contradict the study conducted in a rural area of the department of Vaupés, Colombia, in 2020 that showed no statistically significant differences were observed in the prevalence of TF in children who have a toilet seat in their homes and those who deposit fecal matter between 50 and 200 meters, or more than 200 meters, from the home.^25^ It was evidenced that many people with latrines in their homes still preferred to deposit their excreta in open fields, far from their homes.

This finding showed that children from families who used open defecation (AOR: 2.8; 95% CI: 1.24, 6.1) and outside near the house (AOR: 2.5; 95% CI: 1.1, 6.1) to defecate were 2.75 and 2.5 times more likely to have active trachoma than children living in a household using any form of latrine, respectively. This finding was in agreement with similar studies conducted in Oromia, Grar Jarso District, northern Ethiopia, and Gonji Kolella District, northern Ethiopia.^1^^,^^2^^,^^12^ This finding was also in line with a study conducted in Dera Woreda, northwest Ethiopia, in 2015 (OR: 2.7; 95% CI: 1.5, 4.8), which showed feces around the main house were significantly associated with active trachoma.^13^^,^^27^ The study conducted in Kajiado County, Kenya, in 2017 also came up with the same findings.^24^ This is due to the fact that the bacteria *C. trachomatis* is carried by the fly *Musca sorbens* (eye-seeking fly) from infected eye to human feces because human feces is the most comfortable medium for *M. sorbens* breeding. Then, the fly carries the *C. trachomatis* to a healthy eye. This implies that the proper disposal of human feces would have broken this transmission channel and prevented humans from acquiring active trachoma.

According to the study’s findings, children who did not wash their faces with soap had a nearly 2-fold increased risk of having an active trachoma infection compared with their peers (AOR: 1.9; 95% CI: 1.1, 3.1). This outcome was consistent with research conducted in the Baso Liben District of East Gojjam, Ethiopia, which found that children who did not use soap had a 5.8 times higher likelihood of having an active trachoma infection. A study conducted in rural communities of the Lemo District, southern Ethiopia indicated that the odds of having active trachoma among children who did not use soap during face washing were 2.3 times higher than for children who used soap (OR: 2.3; 95% CI: 1.32, 4.12).^6^^,^^14^^,^^26^ This finding was also in line with a study conducted in Kenya in 2019 that found that children who do not use soap were 15 times more likely to have active trachoma than their counterparts.^3^ This could be justified in that face flies are abundant in trachoma-endemic areas. In this arid climate, they seek moisture in the eyes and mucosal membranes of children. This implies that washing hands and face with soap has been evidenced to be one of the best ways of removing germs and preventing the spread of active trachoma infection.

## CONCLUSION

The prevalence of active trachoma in the Liben District was found to be 29.2%. Prevalence of active trachoma in children aged 1–9 years studied in the Liben District, southern Ethiopia was found to be high, and it is much higher than the WHO trachoma elimination threshold. Being widowed, living with a family who did not have a latrine, open defecation, defecating outside near the house, and not using soap for face washing were factors independently associated with active trachoma. The finding implies that trachoma is a major worry among children aged 1–9 years of the pastoralist community that needs additional consideration from district and zonal health offices, regional health bureau, and different partners who are planning and applying trachoma elimination projects.

Finally, it is advised that this high prevalence of active trachoma demands a comprehensive program that includes each of the four components of the SAFE strategy for controlling and eliminating trachoma. The community has to be educated and mobilized about the value of personal hygiene and environmental sanitation, including the building of latrines and the use of soap for facial cleaning, by the kebele leaders, health professionals, and HEWs. The community should also be more conscious of the needs of widowed household heads. IECWs should focus more on community-based TT screening of adults.

## Supplemental files


Supplemental materials
