# Supplementary material for: Active Trachoma Prevalence and Related Variables among Children in a Pastoralist Community in Southern Ethiopia in 2021: A Community-Based Cross-Sectional Study
Source: Am J Trop Med Hyg. 2023 Jan 9;108(2):252–60. doi: 10.4269/ajtmh.22-0521 (PMC9896328; doi:10.4269/ajtmh.22-0521)
Supplement: Supplementary file 1 [file tpmd220521.SD1.pdf]

CONCEPTUAL FRAMEWORK

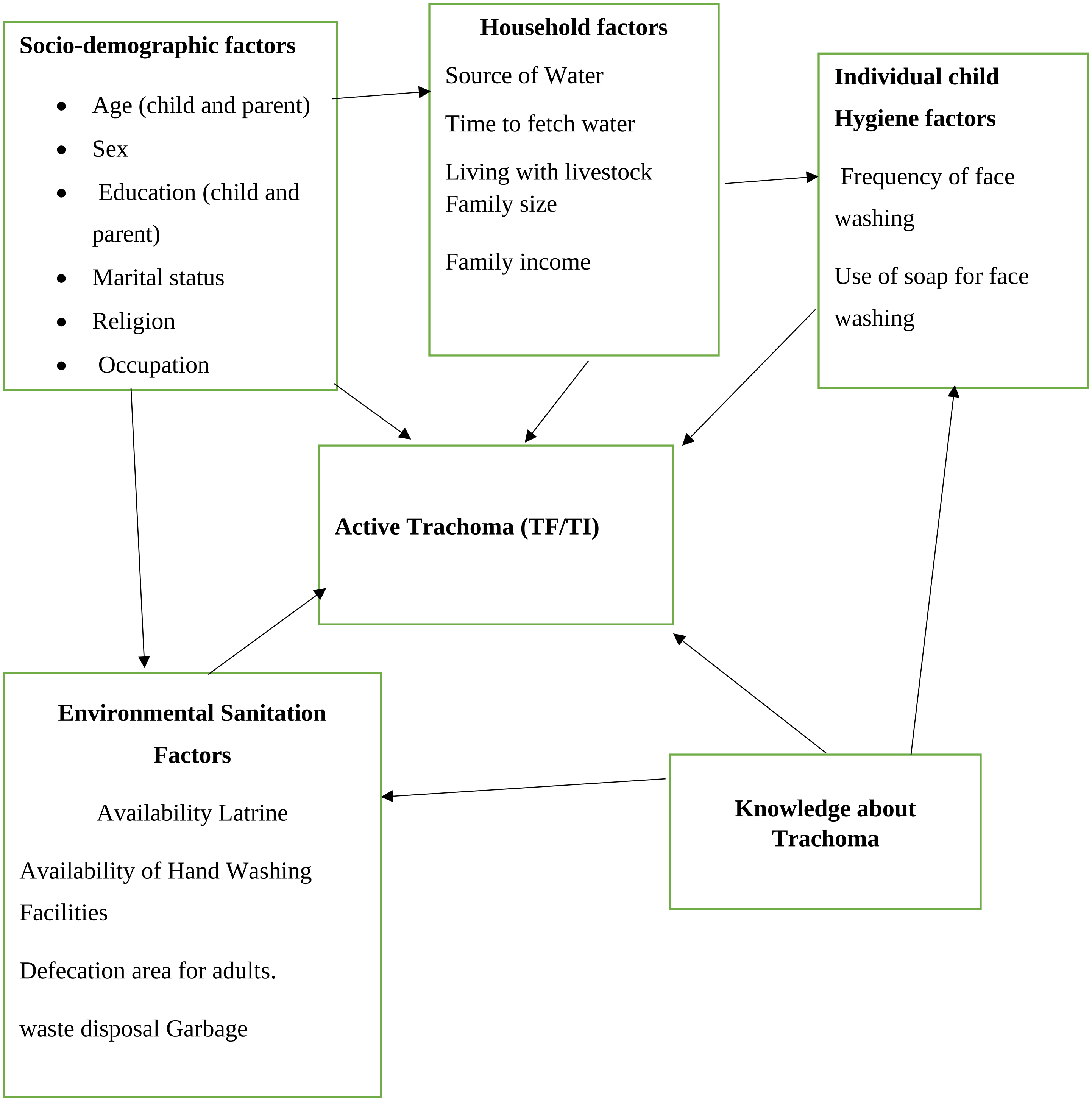

Figure 1 Conceptual framework of factors influencing active trachoma adapted from different literatures, Liban district,2021.

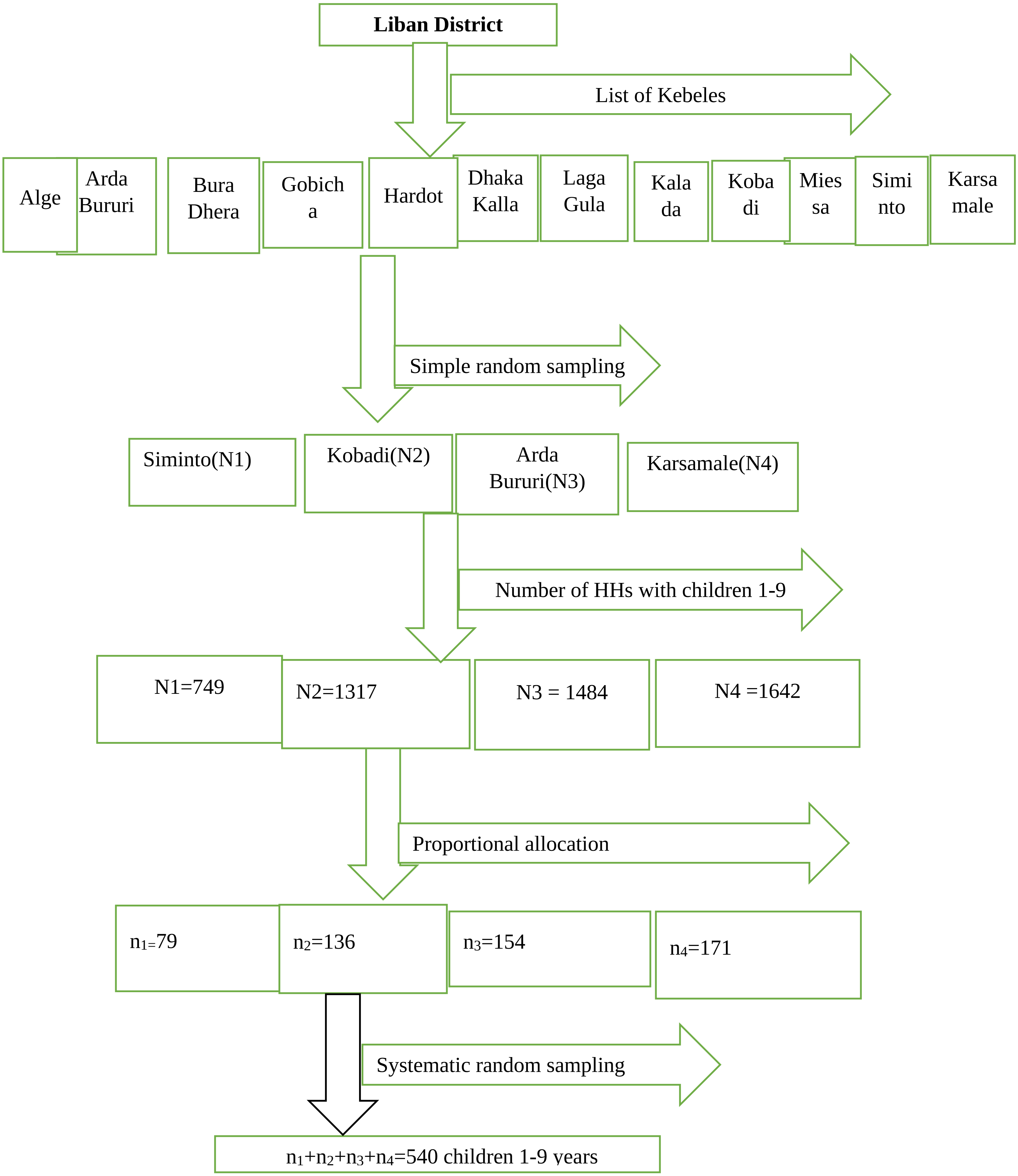

Figure 2 schematic presentation of Sampling procedures, Liban distrct,2021.

I. **Study Questionnaire**

Active trachoma prevalence and related variables among children in a pastoralist community in Southern Ethiopia in 2021: A community- based cross-sectional study.

Part I: Interviewer administered questionnaire

Zone: ___________ District: _____________ Kebele: __________ Village: ___________

Name of Data Collector: __________________ Date: _ _ /_ _ /_ _ (DD/MM/YY)

**No.**	**Questions**	**Answers (Coding Category)**
	Unique Questionnaire No.	
	**Socio-demographic factors**	
1	Age of respondent in years	|___|
2	Sex of respondent	1. Male<br>2. Female
3	Marital status	1. Single<br>2. Married<br>3. Divorced<br>4. widowed
4	Educational status of head of house hold	1. Illiterate<br>2. Can read and write<br>3. Elementary school<br>4. High school & above
5	If the selected respondent is in school or drop out of school, what is the highest grade completed?	|___|
6	Ethnicity	1. Oromo<br>2. Amhara<br>3. Others
7	Religion of head of house hold	1. Muslim<br>2. Orthodox Christian

		3. Protestant<br>4. Wakefata<br>5. Other___________
8	Occupation of head of house hold	1. Farmer<br>2. Merchant<br>3. Daily Laborer<br>4. Employee<br>5.<br>others_______________
9	Age of child	1. 1-4 years<br>**2.** 5-9 years
10	Sex of child	1. Male<br>**2.** Female
11	Educational status of child	1. Too young<br>2. Not enrolled<br>3. Dropped out of school<br>4. Attending school
	**Household Information**	
	**Family size**	
12	How many people live in this household?	|___|
13	Number of children 1-9 years in the household	1. <5<br>2. >5
	**Water supply**	
14	What is the main source of water for domestic use of members of your household?	1. Piped water/public tap<br>2. Protected well/spring<br>3. Unprotected well/spring<br>4. Rain water collection<br>5. Surface water (River/pond)

15	How long does it take to go there, get water, and come back?	1. <15 minutes<br>2. 16-30 minutes<br>3. 31-60 minutes<br>4. >60 minutes
16	Do you have any animals (cattle, sheep, goats, camels)?	1. Yes<br>2. No
	**Environmental Sanitation Factors**	
17	Where do you and other adults in the household usually defecate?	1. Shared latrine with neighbor.<br>2. Private latrine<br>3. Outside near the house<br>4. Use bush or field
18	Ask to see the availability of latrine/toilet.	1 Yes<br>2 No
	Ask and observe the utilization of latrine	1. Yes<br>2. No
19	Observation: Is there a handwashing facility within 15 meters of the latrine/toilet?	1. yes<br>2. No<br>3. Not applicable (no latrine)
20	Observation: At the time of the visit, is water available at the handwashing facility?	1. Yes<br>2. No<br>3. Not applicable (no hand washing facility
21	Observation: At the time of the visit, is soap or ash available at the handwashing facility?	1. yes<br>2. No<br>3. Not applicable (no hand washing facility)
22	Where do you dispose of your garbage?	1. Burning<br>2. Drying

		3. In open field
	**Individual child Hygiene factor**	
23	How often do you wash your child's face?	1. Only occasionally<br>2. At least once per day<br>3. More than once per day
24	Soap use during face washing	1. Yes<br>2. No
	**Knowledge about Trachoma**	
25	Do you know any illness called Trachoma?	1. Yes<br>2. No
26	Do you know sign and symptom of trachoma?	1. Yes<br>2. No
27	What sign and symptom does trachoma show? (Multiple answer is possible)	A. Eye discharge Yes No<br>B. Redness of eye Yes No<br>C. Light intolerance Yes No<br>D Itching Yes No<br>E Irritability Yes No
28	Do you know cause of a trachoma?	1. Yes<br>2. No
29	Do you know consequence of trachoma?	1. Yes<br>2. No
30	Is trachoma preventable?	1. Yes<br>2. No
31	How can we prevent trachoma?	1. Environmental cleanliness<br>Yes No<br>2 Personal hygiene Yes No

		3 Latrine construction and utilization Yes No<br>4 Proper waste disposals<br>Yes No<br>5 Living with livestocks<br>Yes No

Part II: Observation and eye examination

S.No.	Variable Description/Question	Answer/Code
1	Is there ocular discharge in his/her face?	1. Yes<br>2. No
2	Is there a nasal discharge in his/her face?	3. Yes<br>4. No
3	Is there a fly on his/her face, within 3 seconds of observation?	1. Yes<br>2. No
4	Is there sign of active trachoma on eye examination?	1. Yes<br>2. No
5	If Q 4 Yes, specify the stage of trachoma	1. TF<br>2. TI<br>3. Both TF/TI<br>4. TT
6	Is there in turned Eye lash in his/her eye	1- Yes<br>2- NO
7	If Q6 yes,is it rubbing a cornea?	1- Yes<br>2- No
8	Presence of human feces in the compound	1. yes<br>2. No

Thank you for your participation.

I. Verbal Consent

Madda Walabu University, Shashemene Campus, department of Public Health "Active trachoma prevalence and related variables among children in a pastoralist community in Southern Ethiopia in 2021: A community- based cross-sectional study."

Informed Verbal Consent

Dear respondent,

Hello, my name is ________________, I am Health Extension Worker/IECW of Liban Woreda and I am also a member of research team of Madda Walabu University conducting a study on **Active trachoma prevalence and related variables among children in a pastoralist community in Southern Ethiopia in 2021**. I would very much appreciate your participation in this study. I will ask you some questions about your socio demographic back ground, household information, Environmental sanitation, child hygiene, knowledge factors and perform eye examination for one of your Child in the household.

Your honest and genuine response will highly be appreciated and credited as it will enable to make realistic analysis and to propose some very practical suggestions.

The interview and eye examination may take about 30 minutes to complete. Whatever information you provide will be kept strictly confidential and will not be shared with anyone other than my supervisor and any other relevant.

The interview is voluntary. You can refuse to participate in the interview or refuse to answer any questions that you do not want to answer in the middle of the interview. However, your willingness to answer all questions will be appreciated.

Do you have any questions? Do you agree to participate?

Thanks, in advance.

If you have further question or concern you can contact me any time.

Phone :- +251 913285827
